# Assessment of the Adaptive Behavior of Young Children with Visual Impairments in an Early Intervention Service: A Pilot Study

**DOI:** 10.3390/children11101263

**Published:** 2024-10-18

**Authors:** Valerie Caron, Sibilla Badaracco, Geneviève Petitpierre, Saheb Yousefi

**Affiliations:** 1 Department of Special Education, University of Fribourg, 1700 Fribourg, Switzerland; sibilla.badaracco@edufr.ch (S.B.); genevieve.petitpierre@unifr.ch (G.P.); 2 Faculty of Psychology and Educational Science, Allameh Tabataba’i University, Tehran 1489684511, Iran; s_yousefi@atu.ac.ir

**Keywords:** visual impairment, adaptive behavior, communication, daily living, social, motor skills

## Abstract

Introduction: Adaptive behavior, defined as a critical set of skills learned and performed throughout daily life to cope with society’s age-appropriate expectations, is a central concept for people with disabilities in both clinical and research contexts. As AB is an essential component of daily functioning, assessment is necessary both for the diagnostic process and for intervention, as it enables scores to be compared with the developmental norm, identifies strengths and weaknesses of the persons and monitors the progress of interventions. AB assessment is common in children with developmental delays but less common in children with visual impairment (VI). The aim of this study was to evaluate the AB of young children with VI through a pilot study and descriptive data. Methods: The participants were recruited through an early childhood special needs education service specialized in VI in the French-speaking part of Switzerland. Overall, 10 families gave their consent for their child to be assessed using the Vineland Adaptative Behavior Scale-II (VABS-II) completed by their early childhood educator. Results: Globally, the results showed that participants were rated between the Adapted to Moderately High levels. The highest domains were in the areas of communication, daily living skills and socialization. Discussion: The results showed a trend that is superior to previous studies assessing the AB of a similar population. As a result, one obvious perspective would be to adapt the scale to ensure that the items assessed are more consistent with the specificities of their development and the intervention priorities, enabling them to achieve adaptive behavior and independence in carrying out activities of daily living.

## 1. Introduction

The World Health Organization (WHO) (2023) estimates that 2.2 billion people are affected worldwide by visual impairment (VI), which can range from mildly or severely impaired vision to complete blindness [1]. Visual impairment (VI) often has a profound impact on many aspects of the individual’s life, health, participation and performance in daily activities, referred to as adaptive behavior [2]. The impact, however, varies, depending on the severity of the VI, income level, and also individuals’ access to care and services [3]. In children, several areas of development (i.e., cognitive, motor and socio-emotional) could be affected [4]. AB refers to a large set of skills that people learn throughout their life and mobilize to meet social expectations. The assessment of adaptive abilities is as relevant to research as it is to practice [5]. AB enables the situating of people’s abilities in the three domains proposed by the American Association on Intellectual and Developmental Disabilities (AAIDD) [5], namely (1) the conceptual domain: receptive and expressive language, reading, writing, spatial orientation, telling time; (2) the practical domain: activities of daily living, safety, care; and (3) the social domain: interpersonal relations, responsibility, awareness of danger. AB assessment is worthwhile for several practical and theoretical reasons. At the clinical level, AB assessment can be used to refine a diagnosis, such as intellectual disability, and/or to identify priority areas for intervention [6]. Jonker et al. [7] point out that people with limitations in adaptive functioning, but in whom the intellectual disability is not noticeable at first sight, may face the risk of not being recognized as such, with the consequence of being deprived of the support they need. At the system level, assessing adaptive skills may provide a profile of the recipients of a service, as well as up-to-date pictures of their needs if the assessments are repeated on a regular basis. On a scientific level, AB assessment can be used to describe and monitor the evolution of participants. In a broader perspective, it might also be used to understand, for instance, the role of living contexts in developmental trajectories [8]. This can be achieved by, for example, examining the adaptive profile in relation to the density of facilitators and obstacles in the environment of a sample, comparing the overall versus the domain-specific adaptive dimensions, and their variability, to study the effects of specific interventions or support. In this case, international comparisons could be of great interest.

Although several measurement instruments have been developed to assess AB, only a few have good psychometric properties [6]. Among the recommended instruments, the Vineland Adaptative Behavior Scale (VABS-I, VABS-II and VABS-III) aim to assesses AB based on five main domains, namely (1) communication; (2) activities of daily living; (3) socialization; (4) motor skills; and (5) behaviors, as well as fourteen subdomains [9,10,11]. Analysis of the results indicates the level of adaptation per domain, as well as the overall level of adaptation expressed by the composite score on a five-level scale (low, moderately low, adequate, moderately high or high). From a theoretical point of view, and contrary to previous definitions, physical or motor skills and behaviors are no longer considered as components of adaptive behavior [6]. The consequence of this is that, in the VABS, which devotes a scale to each of these dimensions, the results of the scales concerned must be analyzed separately and not as a component of the adaptive profile. A second recommended scale is the Adaptive Behavior Assessment System (ABAS-II and ABAS-III), which assesses AB according to the three main domains of the AAIDD model and ten sub-skills [12,13]. The scores obtained define whether the person is average or below average. These two scales assess AB indirectly via questionnaires for parents and/or teachers. They have been validated in samples of people with different types of disabilities, including but not limited to VI. Their use with different clinical populations is therefore recommended (e.g., with people with autism spectrum disorders, intellectual disabilities, or sensory impairments). Currently, there is no recommendation in favor of either of these two scales for use with a population with special needs [14]. Recently, a few instruments proposing a contemporary version of the assessment of adaptative skills for youth and those with VI has been developed [7,15]. However, the items are not integrated in the AAIDD framework using three domains.

Although frequently used in the field of intellectual disability, AB has been less studied in the population with VI [3]. In a previous scoping review [16], the authors identified only nine studies dedicated to the AB of children and adolescents with VI aged to 2- to 20-years-old in articles published between 1984 and 2021. Nevertheless, the quality assessment of the identified studies revealed a mean score of 67.8%, i.e., moderate to good methodological quality. The nine studies together included 206 participants with VI aged 4.6 to 19.6 years (*M* = 9.16 years), assessed with VABS-I, VABS-II or ABAS-II. Among the nine articles selected for this review, three studies included pre-school participants (*n* = 4 participants, mean age 4 years and 7 months) [17,18,19]. Two children were described as partially sighted and two as completely blind, and all had been assessed through the first version of the VABS [9]. The mean quality score of the three studies, all case series, was 43.3%, corresponding to low to moderate quality. Several main trends emerged from this review. For conceptual skills, the main trend was toward an Adapted level; for practical skills as well as for social skills, the results were predominantly at a Moderately Low level. Finally, for motor skills, the level of adaptation was mainly Moderately Low to Low. Globally, the review highlighted an overall index of AB represented by the VABS as Moderately Low. These findings indicate that the children with VI assessed have a lower level of functioning than their peers, implying a significant need for assistance in carrying out activities of daily living in the various AB subdomains.

Nevertheless, the findings drawn that participants with VI showed that they scored globally lower than their sighted peers in all areas of AB; however, they also showed intra- and inter-individual heterogenous differences [16]. The conclusions highlighted different strengths in the studies included, such as the use of standardized instruments, but also indicated weaknesses, such as the very small sample sizes (1 or 2 participants) and using a single-case design.

### Children and Adolescents with VI in Switzerland

In Switzerland, the number of children and adolescents with VI is estimated at 1639, which is about 0.17% of the compulsory school age population [20], and most of them receive or have received appropriate intervention and/or support, sometimes from an early age. The Pedagogical Centre for Students with VI is a service provider operating in French-speaking Swiss regular or special educational contexts, such as home, school or vocational training centers for children and young people between the ages of 0 and 18. The educational services offered during the pre-school period include an evaluation of functional vision, early childhood special needs stimulation for the child, familial support and/or child support in inclusive educational settings, such as day-care centers. Annually, between 50 and 60 children receive early intervention services from specialized VI services. There is no record of the number of cases that do not receive services, but these are referred to this service systematically by the ophthalmology department, which establishes low-vision diagnoses. Support is usually provided by a multidisciplinary team, but standardized tools are rarely used [21,22]. Given the support needs of very young children with VI in many areas of life and the lack of scientific knowledge about AB in diagnosis and follow-up interventions, assessing the AB profile of young children with VI in a larger group seems important. Therefore, the aim of this study is to assess the AB of children with VI, aged between 2 and 4 years, attending the main low vision service in French-speaking Switzerland.

## 2. Materials and Methods

The survey was conducted in an early intervention center specializing in visual disorders. The recruitment of participants was carried out by the service itself. To be eligible for the study, participants had to be receiving educational services from the service after a diagnosis of VI and be aged between two and four years—that is, younger than the start of compulsory schooling in Switzerland. No exclusion criteria were retained for possible comorbidities. With the agreement of the principal, the early childhood special needs educators working in the service were invited to participate in the study through a written, simple and concise document explaining the objectives of the research project. The call was followed by a face-to-face information session about the Vineland-II (French version). For the version translated into French, the fidelity was judged to be good for children aged 1 to 6, with average scores for the subdomains (0.9). The internal consistency was also rated as good to excellent [23]. Nevertheless, the validation sample for the instrument included children with VI but not only them. The authors recommended the tools for children with different disabilities but not specifically for only VI as the content is not validated for this specific population.

Parental consent was requested by means of a written form that allowed, or did not allow, the early childhood special needs educators to complete the questionnaire for their son or daughter. The educators were invited to complete the questionnaire alone or in collaboration with the parents. Once completed, the anonymized VABS-II questionnaires were returned to the research team by mail or in electronic format. To preserve the anonymity of the participants, each participant was given a numerical code between 0 and 10.

### 2.1. Participants

Of 30 children aged between 2 and 4 years old who met the inclusion criteria, 10 participants participated in the study (3 participants were diagnosed as blind and 7 with low vision functioning). The participants were 6 boys and 4 girls aged between 24 and 49 months (*M* = 36 months). The severity of the VI, as well as the exact diagnosis, and information about potential comorbidities could not be obtained. The data were collected at the end of the year in which the children did not received any specific intervention to improve their AB. They received a comprehensive intervention provided by the early childhood program but not specifically to develop adaptative skills. Table 1 presents the characteristics of the sample.

### 2.2. Measure

The Vineland Adaptive Behavior Scale-II (VABS-II) was used to assess the participants’ AB; more specifically, the French version survey form was used [23]. The VABS-II was selected because it is a commonly used standardized hetero-reported instrument with good psychometric properties that can be used throughout the lifespan [6]. The instrument provides an overall level of adaptation for each child (translated by the Vineland Adaptation Behavior Composite [VABC] index), as well as a level of adaptation for the four domains and 11 subdomains: (1) communication (receptive, expressive, written); (2) daily living skills (DLS) (personal, domestic, community); (3) socialization (interpersonal relationships, play and leisure, coping) and (4) motor skills (gross and fine). The adaptive levels (ALs) are as follows: Low, Moderately Low, Adequate, Moderately High, or High, along with a typical corresponding age. Information regarding the potential occurrence of internalized, externalized, other and critical behaviors was also collected through the VABS section. For each behavior assessed, the possible answers were: (0) behavior is not present; (1) behavior is sometimes present/partial; or (2) behavior is usually present.

### 2.3. Data Coding and Analysis

The raw scores were first calculated manually for each AB domain. Compilation of the raw scores was performed with the Vineland-II software. The participants’ scores were then converted into v-scores, standardized scores allowing a level of adaptation for each domain and subdomain, an equivalent age, and an overall composite level of adaptation (VABC) to be obtained. Data reflecting the problem behavior domain were scored and analyzed manually. The final data were compiled in an Excel file and the results were synthesized in tables or figures. For the results presentation, the test scores were grouped into bands, with qualitative descriptors (Adaptative Level) assigned to the score bands, for easier description.

## 3. Results

### 3.1. Vineland Adaptive Behavior Composite (VABC)

Of the ten participants, one child scored Low, two scored Moderately Low, six scored Adapted, one scored Moderately High and no participants scored High. Although the participants were distributed across all the VABC levels, the trend seems to be consistent with an Adapted overall level of adaptive functioning. Table 2 presents the overall level of adaptive functioning for each participant, as well as their adaptive levels by domain and overall level of adaptive functioning.

### 3.2. Communication Domain

**Figure 1:**
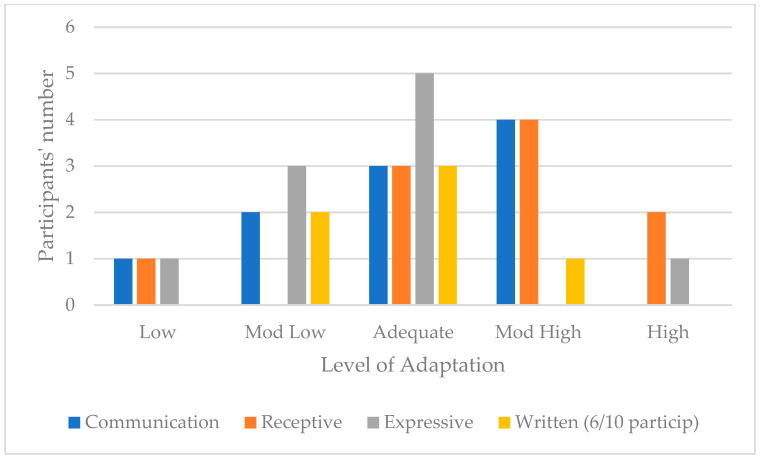
Communication domain scores across the sample.

Of the ten participants, one scored Low, two scored Moderately Low, three scored Adequate, four scored Moderately High and none scored High. The trend is toward a fairly high/adequate communication adaptive level. Figure 1 illustrates the distribution of participants and their adaptive level in the three communication subdomains. Regarding receptive communication, the trend is toward a moderately high/adequate receptive adaptive level. In expressive communication, the trend is toward an adequate/moderately low expressive adaptive level, and toward an adequate/moderately low written adaptive level in written communication. For this subdomain, only six participants were considered because their young age did not allow the last four to be able to write. Figure 1 present the communication domain scores across the sample.

### 3.3. Daily Living Skills Domain

**Figure 2:**
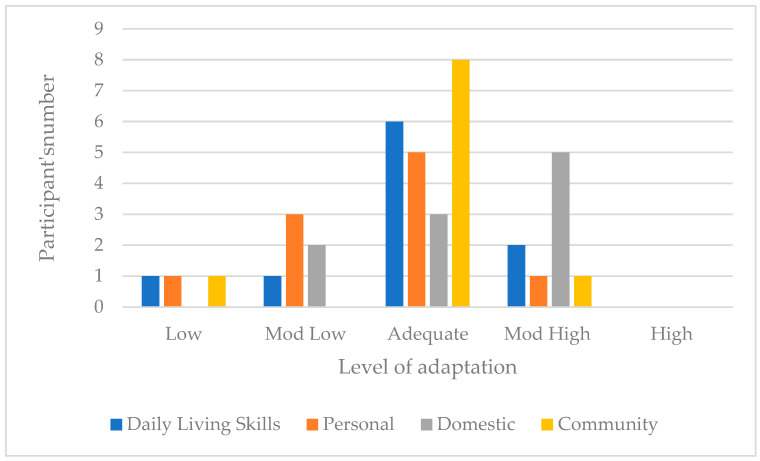
Daily living skills domain scores across the sample.

For this domain, one participant scored Low, another Moderately Low, six Adequate, two Moderately High and no participants scored High. The trend is toward an adequate daily living skills adaptive level. Figure 2 illustrates the distribution of participants and their adaptive level in the three DLS subdomains. Regarding personal skills, the trend is toward an adequate/moderately low personal adaptive level. Regarding domestic skills, the trend corresponds to a moderately high/adequate domestic adaptive level and to an adequate adaptive level, achieved by 80% of the sample, for community daily living skills. Figure 2 present the daily living skills domain scores across the sample.

### 3.4. Socialization Domain

**Figure 3:**
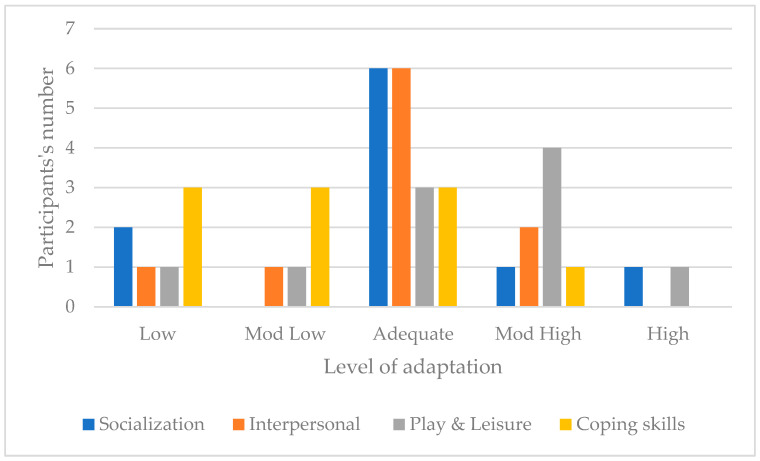
Socialization domain scores across the sample.

For this domain, two participants scored Low, none Moderately Low, six scored Adequate, one Moderately High and one scored High. The trend is toward an adequate socialization adaptive level. Figure 3 illustrates the distribution of participants and their adaptive level in the three socialization subdomains. Regarding interpersonal relationships, the trend is towards an adequate interpersonal adaptive level. The trend is toward a moderately high/adequate level for play and leisure skills, and toward an adequate/moderately low/low level in the coping skills subdomain. Figure 3 presents the socialization domain scores across the sample.

### 3.5. Motor Skills Domain

**Figure 4:**
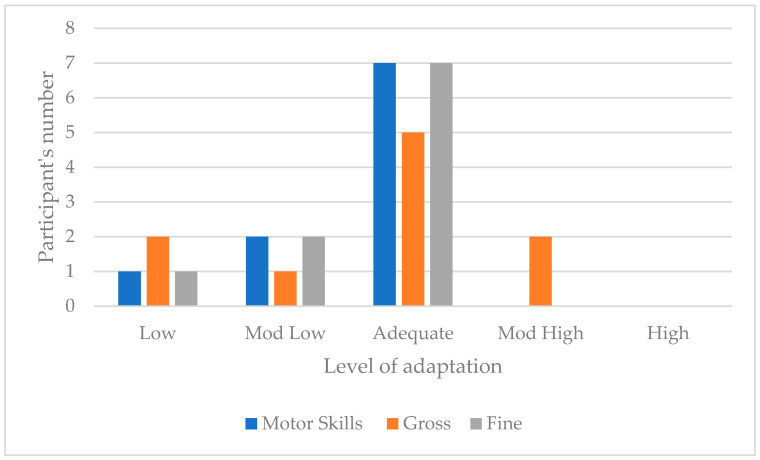
Motor skills scores across the sample.

For this domain, one participant scored Low, two obtained Moderately Low, seven Adequate and none obtained a Moderately High or High level. The trend corresponds to an adequate motor skills adaptive level. Figure 4 illustrates the distribution of participants and their adaptive level in the two motor skills subdomains. Regarding gross motor skills, the trend corresponds to an adequate gross motor skills adaptive level and to an adequate level in the fine motor skills subdomain. Figure 4 present the motor skills domain scores across the sample.

### 3.6. Behaviors

For internalized behavior, the most frequent was sleep disturbance (*n* = 4); for externalized behavior, disobedience and stubborn/sulking was reported (*n* = 5); and for other behavior, bed wetting was the most frequent (*n* = 5). The complete results for the behavior section are presented in the Supplementary Materials.

## 4. Discussion

Although the sample size was smaller than expected, the results are interesting in several ways. With respect to conceptual skills, defined by the AAIDD by receptive and expressive language, reading, writing, spatial orientation, telling time [4], the results showed that the participants scored consistently with Adapted to Moderately High levels. This level of performance is higher than that reported in studies of preschool children with VI published to date with a single-case design (*n* = 3 participants) [17,18,19] but similar to that obtained in a single study (*n* = 1 participant) [17]. This result may be explained by the participants’ young age. It is also possible that, due to needs in other essential areas, the children in the sample, even the older ones, did not have the opportunity to develop their writing skills, or that the educational focus was on mastering the prerequisites for writing, which are considerably more demanding for children with VI due to the lack of visual feedback on both gestures and posture. As the scientific literature reported that the greatest communicative delays in the field of VI appear during the early stages of development as language acquisition is more dependent on sight [24], it is therefore not surprising that, among our sample, the children with the lowest adaptive level in the field of communication were also part of the youngest age range.

However, it is also possible that the conceptual skills for early childhood in those with VI could be different as they do not learn in the same way as sighted children. For example, the development communication [24] and sensory-motor skills could be developed in a different way (i.e., better sound localization, spatial hearing, manipulatory skills [25]. In this context, the use of the VABS developmental milestones may not be suitable for assessing the development of children with VI, since they develop differently from sighted children. For intervention, they also need multi-sensory programs adapted to their specific needs [26,27,28,29]. Many authors have suggested that sensory efficiency skills (auditory, tactile) are more important for these young people, enabling them to achieve the independence to master different skills such as walking with cane, writing or reading in braille [28,29,30].

Regarding practical skills, defined by the AAIDD activities of daily living, safety, and care [5], the main trend of the results shows the Adequate adaptive level of the children in the sample. This good autonomy level in the daily living skills domain contrasts with the results of previous studies, which have shown weaker results, indicating lower abilities during pre-school age [17,18,19], childhood [31], as well as adolescence [32,33,34] and adulthood [35]. The Adapted level during the pre-school period is a good sign, but it is not enough, and it is important to remain vigilant about learning in this area. As for the conceptual skills, during early childhood, the practical skills could be different for children with VI and their independence may depend more on orientation and mobility skills such as standing alone, using a pre-cane, holding the cane or avoiding obstacles during walk [36,37,38,39]. From this perspective, a more accurate assessment of practical skills from an early age, with a tool adapted for VI [37], as well as the continuation of teaching throughout the school years, seems fundamental to optimize the practical skills of children with VI [40]. Indeed, from the school period onwards, practical skills are often neglected in education in favor of academic learning and are not always well integrated into the school curriculum by implementation of the expanded core curriculum [41]. Some authors mentioned that teachers may experience challenges and barriers in implementing expanded core curriculum teaching rather than the school curriculum [41]. For young people with VI, these skills are, however, critical, as they will impact their daily lives, mobility independence and future work-related skills [40,41,42].

With regard to social skills, defined by the AAIDD by interpersonal relations, responsibility, and awareness of danger [4], as with the previous two domains, the trend in our results indicates an Adequate level that is higher than those obtained in other studies, which have indicated a deficit during the pre-school period [17,18,19] or during adolescence [30,33,34]. Like practical skills, social skills could be weaker in individuals with VI than in their sighted peers, as these skills are often acquired through imitation and observation [43]. However, social skills deficits in this domain can negatively impact the individual’s social participation as well as their social support [44,45]. Therefore, because young people with VI are partially or completely deprived of imitative and implicit peer learning opportunities to acquire social skills, these skills must be taught explicitly with different strategies, such as verbal instruction/feedback, prompting, role-playing, modeling or peer-mediation [46].

With respect to motor skills, which are not included in the AIDD model [5] but assessed in the motor domain of the VABS questionnaire, the trend that emerges from the results is Adequate. Like the previous three domains, our results are higher than those obtained by other authors who obtained low scores demonstrating weaker motor abilities in pre-school children with VI compared to sighted children [17,18,19]. Moreover, many authors indicated that motor skills deficits are maintained and deepened in young people with VI during childhood and adolescence [47,48]. The levels of vision and physical activity are identified as two predictors of performance in motor skills [48,49]. Since the motor skills assessed in the VABS follow normal development, it is possible that the milestones assessed are not the right ones for children with VI, since they often follow different developmental stages [36]. In addition, the weaknesses identified in the VABS results should also be considered in relation to orientation and mobility skills, such as the development of sensory efficiency for gross motor skills as early cane skills, echolocation skills [37] as well as fine motor skills (i.e., pre-braille skills, tactile readiness and auditory skills) [28]. 

## 5. Conclusions

The aim of this study was to assess the AB of young children with VI using the standardized VABS-II scale using a pilot study with a small sample. All the participants were recruited in the early childhood low vision service in French-speaking Switzerland. The participants’ profile was characterized by the communication domain being the strong point (Adaptation Level is Adapted/Moderately High), compared to the other domains being weaker, although they were all considered Adapted.

### 5.1. Limitations

The aim of this pilot study was to assess the AB of young children attending a low vision service. Consequently, as a pilot study, it has a number of limitations that point to important avenues for future research. Among the most important limitations, the voluntary nature of this study enabled modest and descriptive data to be collected from a very small sample, and a lack of information on the children’s comorbidities prevented the results from being generalized to a similar population. Nevertheless, other studies on the topic had sample sizes limited to one to three participants, and the present sample size (*n* = 10) is only slightly larger. This is due to the fact that, for obvious and legitimate ethical reasons, the principle of voluntary participation by families has been fully respected. All the families benefiting from the services of the early childhood special needs service in VI in the district of Lausanne (the chosen geographically defined area) were contacted, but unfortunately, only a third agreed to take part in the study. This situation is not unusual in research involving very young children, many of whom have just been diagnosed. On the one hand, participating in research is often not the priority for their families at this time of their lives. On the other hand, agreeing to take part in research generally implied that the diagnosis has been “integrated” by parents, if not accepted. For these different reasons, voluntary participation in this study remains a selection bias that prevents the generalization of the results. Previous research has pointed out that voluntary samples may be biased toward families that are already highly dedicated and thus seek help in an early intervention center [50]. In this research, this kind of bias seems limited as families of children with visual needs are systematically referred to the early childhood visual needs service by the ophthalmology department of the Vaud region hospital center, which establishes low-vision diagnoses, and there is no record of the number of cases that do not receive services. Annually, between 50 and 60 children between 0 and 6 years old receive early intervention services from the early childhood visual needs service in the canton of Vaud. This reality does not change the fact that the results cannot be generalized, but it does show the conditions of research in this age group. Moreover, another serious limitation includes the lack of information on potential comorbidities as well as the imprecision of the level of visual impairment (i.e., acuity, field), which was not available for the researchers. These results could open up a clinical perspective for systematizing information on visual impairment in children’s files.

In addition, the use of an instrument whose validation sample included people with VI, but not only them, made it possible to document the children’s profiles but also to observe that the instrument was not totally adapted to this population. As some missing questions were interpreted with a score of 1, the corresponding procedure provided in the user manual, these missing data could have distorted the results. Indeed, respondents found some items were not precise enough to capture the skill subtleties of very young children with VI. As a matter of fact, the wording of certain items, written for a typical and/or sighted population, gave rise to some uncertainty on the respondents’ part as to how to code the responses. Therefore, some respondents added details by hand, mainly concerning the way in which the behavior was expressed in a particular child. For example, the item of walking did not reflect the fact that some children walked independently with a cane, or the item of reading and writing did not reflect braille reading and/or assistive technologies. This qualitative information could not be considered in the interpretation of the results. Consequently, this lack of specificity in the items assessed in the VABS-II represents another important limitation of this study and a major perspective for future research.

### 5.2. Implications for Practice and Research

Prior to the present study, no studies had documented the AB of young children with VI in Switzerland and there were very few such studies in the scientific literature. The few studies on the subject have used single-case designs for young children or groups of adolescents and adults. Considering the scientific literature on the development of children with VI and the conclusion of this study, it seems clear that they adopt different pathways from sighted children. Consequently, despite important limitations, this study contributes and paves the way for various avenues of future research: first, evaluation of the AB of children and adolescents with VI with a large sample, as well as longitudinal studies, seems essential to monitor their development and the progress of rehabilitation services. For this purpose, further study of the adaptive functioning of young people and adolescents with VI would make it possible to define possibly different milestones that consider their developmental specificities and also intervention priorities (i.e., development of sensory skills, writing in braille, walking with a cane, orientation and mobility skills). In this context, a final avenue for future research would be to pursue reflection on a definition of AB for people with VI, so that assessments are consistent with the priority skills enabling them to optimize their autonomy in the skills of daily living. To this end, adaptation and content validation using a Delphi study [51,52] would seem to be a prerequisite for validating the psychometric qualities. 

For practitioners, these results could be helpful in guiding avenues of intervention in early intervention to prevent weaknesses in the AB of children with VI. According to the three domains of AB, it is therefore possible to identify many skills to develop in early intervention. Firstly, for conceptual skills (referring to receptive, expressive language, reading, writing, spatial orientation), the development of sensory efficiency is crucial for all early learners with VI (i.e., auditory, tactile, olfactory). The parents and practitioners could teach these skills with hands-on experience, encouraging exploration with a variety of sensory material and using verbal descriptions of activities and the environment. Secondly, practical skills (referring to daily living skills, safety and care), the development of body concepts, spatial awareness, crawling skills, mobility and walking with a pre-cane could be taught in natural environments through routines [28,37]. Finally, for social skills (referring to interpersonal relation, awareness of danger), the early learners with VI need to be encouraged to explore their environment, to play with a variety of sensory toys and to need clear description of their environment to feel safe to explore [28].

## Figures and Tables

**Table 1:** Characteristics of the sample.

Participant	Age (Months)	Sex	VI
1	42	B	LV
2	36	B	LV
3	41	B	LV
4	24	G	LV
5	35	B	LV
6	28	G	B
7	38	B	LV
8	27	G	LV
9	49	G	B
10	43	B	B

Note. Boy (B), Girl (G), Visual Impairment (VI): Low Vision (LV), Blindness (B).

**Table 2:** Levels of adaptative behavior by domain and VABC overall level.

Participant	Communication	Daily Living	Socialization	Motor Skills	VABC
1	Adapted	Adapted	Adapted	Mod. Low	Mod. Low
2	Mod. High	Mod. High	High	Adapted	Mod. High
3	Adapted	Adapted	Mod. High	Adapted	Adapted
4	Mod. Low	Adapted	Adapted	Adapted	Adapted
5	Low	Low	Low	Low	Low
6	Adapted	Mod. Low	Adapted	Adapted	Adapted
7	Mod. High	Mod. High	Adapted	Adapted	Adapted
8	Mod. Low	Adapted	Low	Mod. Low	Mod. Low
9	Mod. High	Adapted	Adapted	Adapted	Adapted
10	Mod. High	Adapted	Adapted	Adapted	Adapted

## Data Availability

The original contributions presented in the study are included in the article/Supplementary Material, further inquiries can be directed to the corresponding author.

## Supplementary Materials

The following supporting information can be downloaded at https://www.mdpi.com/article/10.3390/children11101263/s1, Table S1: Frequency of internalized and externalized behaviors reported in the sample (*n* = 10); Table S2: Frequency of others and critical behaviors reported in the sample (*n* = 10).
