# Supplementary material for: Assessment of the Adaptive Behavior of Young Children with Visual Impairments in an Early Intervention Service: A Pilot Study
Source: Children (Basel). 2024 Oct 18;11(10):1263. doi: 10.3390/children11101263 (PMC11506584; doi:10.3390/children11101263)
Supplement: Supplementary file 1 [file children-11-01263-s001.zip › children-3156113-supplementary.pdf]

Supplemental Material

Table S1

Frequency of internalized and externalized behaviors reported in the sample (n=10)

	Frequency	Not present (Score 0)	Partially present (Score 1)	Usually present (Score 2)
**Internalized**				
1. Is dependent on adults	2		1	1
2. Avoids others	0			2
3. Feeding disorders	2		2	
4. Sleep disorders	4		1	3
5. Refuses to go to school or work	1		1	
6. Anxiety	2			2
7. Cries and/or laughs inappropriately	2			2
8. Poor eye contact	1		1	
9. Is sad inappropriately	0			
10. Avoids social interaction	1		1	
11. Lack of energy or interest	0			
**Externalized**				
1. Is impulsive	3		3	
2. Tantrums	4		1	3
3. Disobedience	5		5	
4. Bullying	2		2	
5. Is insensitive	0			
6. Lying, cheating, stealing	1		1	
7. Is physically aggressive	3		3	
8. Stubborn, sulking	5		1	4
9. Makes inappropriate comments	2		2	
10. Behaves inappropriately under the influence of others	2		2	

Table S2

Frequency of others and critical behaviors reported in the sample (n=10)

	Frequency	Not present (Score 0)	Partially present (Score 1)	Usually present (Score 2)
**Other Behaviours**				
1. Thumb or finger sucking	1		1	
2. Wetting the bed or wearing nappies at night	5		1	4
3. Acts familiarly with strangers	3		3	
4. Nail biting	3		1	2
5. Has involuntary repetitive movements	0			
6. Grinds teeth day and night	1		1	
7. Attention difficulties	2		2	
8. Is more agitated than people of the same age	2		1	1
9. Unauthorized or inappropriate use of material	1		1	
10. Using profanity and swearing	1			1
11. Running away	0			
12. Misses school or work for no reason	0			
13. Ignoring others around	0			
14. Uses money inappropriately	0			
15. Uses drugs or alcohol at school or at work	0			
**Critical Items**				
1. Inappropriate sexual behaviours (i.e. undressing, masturbation)	0			
2. Obsession with certain objects or activities (stereotyped use of gestures, words or objects)	0			
3. Makes incoherent statements/comments	0			
4. Strange mannerisms (i.e. stereotyped gestures)	1			
5. Systematically prefers objects to people	0			
6. Self-injurious behaviour (i.e. head banging, biting)	1		1	
7. Destroys the property of others	1		1	

8. Incoherent speech (i.e. talks to themself, stereotyped speech)	0
9. Is not aware of their environment, does not react to what is going on around them	0
10. Rocking back and forth	0
11. Has unusual fear of noises or objects	0
12. Remembers situation in detail many years later	0
13. Is unable to complete a full day at work/school due to chronic pain	0
14. Is unable to complete a full day at work/school due to psychological symptoms	0
